# Effects of Oral Commensal Streptococci on *Porphyromonas gingivalis* Invasion into Oral Epithelial Cells

**DOI:** 10.3390/dj8020039

**Published:** 2020-05-02

**Authors:** Alyssa N. Hanel, Hannah M. Herzog, Michelle G. James, Giancarlo A. Cuadra

**Affiliations:** 1 Department of Biology, Muhlenberg College, 2400 W. Chew Street, Allentown, PA 18104, USA; ah3573@cumc.columbia.edu (A.N.H.); hmherzog@muhlenberg.edu (H.M.H.); mgjames@muhlenberg.edu (M.G.J.); 2 College of Dental Medicine, Columbia University, 622 W 168th St, New York, NY 10032, USA

**Keywords:** oral, commensal, streptococci, *P. gingivalis*, invasion, protection

## Abstract

The objective of this study was to determine if the interaction between common oral commensal bacteria and oral epithelial cells would provide protective effects against the invasion of periodontopathogen *Porphyromonas gingivalis*. Oral epithelial OKF6/Tert cells were used in co-cultures with *Streptococcus gordonii*, *Streptococcus oralis*, *Streptococcus mitis*, and *Streptococcus intermedius*. The viability of OKF6/Tert cells following a bacterial challenge was evaluated by trypan blue exclusion. The adherence of commensal species was determined by CFU counts. *P. gingivalis* invasion in OKF6/Tert cells was assessed before and after exposure to commensal species according to CFU counts. Viability assays show that only *S. gordonii* and *S. intermedius* display low toxicity toward OKF6/Tert cells. Both commensals adhere to OKF6/Tert cells at an average ratio of 1 CFU to 10 cells. *P. gingivalis* invasion into host cells is significantly reduced by 25% or 60% after exposure to *S. gordonii* or *S. intermedius*, respectively. The results suggest that these commensal species bind to host cells and diminish *P. gingivalis* invasion. This is important in the context of periodontal disease since *P. gingivalis* primarily acts on the host by invading it. Therefore, efforts to decrease invasion will eventually lead to future therapies harnessing the mechanisms employed by oral commensal bacteria.

## 1. Introduction

Periodontal disease (PD) is an oral inflammatory condition induced by the diffusion of bacteria into the epithelium surrounding the tooth. PD affects 47.2% of adults over the age of 30 in some capacity, and approximately 70.1% of adults 65 years of age and older have a form of PD [1]. This is exceedingly important, as the number of adults aged 65 and older is increasing markedly and periodontal health is a key contributor to systemic health [2]. PD can cause tooth loss, pain, discomfort and difficulty speaking, chewing and swallowing, which could ultimately lead to poor nutrition [2]. The first stage of PD is caused by a disruption of bacterial homeostasis in the areas that surround and support the tooth, leading to the inflammation and infection of the gums, or gingivitis [3]. The inflammation associated with gingivitis and periodontitis can lead to a proliferation and dilation of the vasculature, which provide a greater opportunity for oral bacteria to enter the bloodstream. While this bacteremia is often transient, it has the potential to reach a variety of target organs [4]. Bacteria that originate in the oral cavity are also associated with several severe systemic conditions including cardiovascular disease [5,6], respiratory infections [7], arthritis [8], diabetes [9,10], and Alzheimer’s disease [11,12].

The oral cavity contains a rich microbiota which, in healthy individuals, remains in homeostasis. The health of periodontal tissues is determined by the composition of this microbiota and the antagonistic and synergistic relationships therein [13]. Pathogenic bacteria within this microbial environment lead to tissue destruction by subverting the host immune response and disrupting the microbial–host homeostasis [14]. Gram-negative anaerobes, most notably *Porphyromonas gingivalis*, are found in large quantities in the subgingival sulcus of patients suffering from periodontitis [15]. Growing evidence suggests that these pathogens have an exceedingly complex relationship with both the host [16] and the nearby commensal microbiota [17,18]. In healthy individuals, several species of streptococci take part in the early colonization of the oral cavity by inhibiting the growth of pathogenic bacteria [19,20]. For instance, *Streptococcus gordonii*, which is regarded as a species of commensal bacteria, is found more frequently in healthy tissue than in areas affected by PD. This commensal species does not alter transcription in oral epithelial cells as significantly as pathogenic strains [21,22]. *S. gordonii* is one of the first species to re-colonize after brushing or antibacterial rinses, which makes it a candidate for the exclusion of pathogenic microbes from tooth surfaces. In fact, oral streptococci constitute over 80% of early oral biofilm and plaque [23]. Commensal streptococci include several groups with multiple species. Examples of these groups and species are the Anginosus group (*Streptococcus intermedius*), Sanguinis group (*Streptococcus gordonii*), and Mitis group (*Streptococcus mitis* and *Streptococcus oralis*) [24]. The four species mentioned have been found to be ubiquitous in healthy and PD-affected individuals [25].

*P. gingivalis* has been studied extensively in the context of its role in the progression of PD. This pathogen is known to adhere to and invade both monolayers and multilayers of primary gingival epithelial cells (GEC) [26,27,28,29] The adherence of *P. gingivalis* is mediated by the major fimbriae, a key virulence factor [30]. Fimbrillin (FimA), a subunit of these filamentous components, interacts with both the host tissue and other bacteria to allow for association [30]. β1 integrins have been identified as epithelial cell cognate receptors for these *P. gingivalis* fimbriae [29]. After *P. gingivalis* initially adheres, with the predominant adhesins being the major fimbriae, an integrin-associated signaling cascade is initiated. Actin microfilaments and microtubule structures in the cell accommodate the entry of the bacteria. This, along with the recruitment of lipid raft components and host-cell signaling events, is required for the internalization of *P. gingivalis* [15]. Once *P. gingivalis* invades, several virulence factors including FimA, RgpA/B and Kgp show decreased expression, and stress-associated proteins such as peroxidases and heat shock proteins are upregulated [15]. These changes result from a need for *P. gingivalis* to adapt to the intracellular environment.

ArcA is a conserved gene found in several oral commensal species and encodes for arginine deaminase [31]. Some studies have investigated ArcA as a factor that potentially inhibits the interactions of *P. gingivalis* with the host tissue, disrupting the progression of periodontal disease. For instance, *Streptococcus cristatus* ArcA interferes with the colonization of *P. gingivalis* in mice [32]. Similarly, arginine deaminase of oral streptococci like *S. cristatus* and *S. intermedius* downregulates the expression of FimA in *P. gingivalis,* which allows for biofilm formation [13,31,33,34]. In addition, commensal bacteria including *S. gordonii*, *S. mitis* and *S. intermedius* were capable of antagonizing the growth of pathogenic species including *P. gingivalis* [20,35]. H_2_O_2_ released by these streptococci appeared to be the most prevalent antimicrobial substance that inhibited the growth of the pathogens [20,36].

The invasive capabilities of commensal streptococci have not been investigated to a great extent. *S. intermedius* interacts with and, to a certain extent, seems to invade hepatic cell models [37]. While *S. gordonii* is known to invade endothelial cells [38], it does not exhibit detectable invasion of gingival epithelial cell (GEC) multilayers [26]. Previous research suggests that *S. gordonii* has the ability to invade human aortic endothelial cells [38], but to date, there is scant research regarding the invasion of *S. gordonii* or any other commensal strain into oral epithelial cells. Conversely, *S. gordonii* tends to induce an immune response in GECs, leading to the secretion of IL-1ß, IL-6, IL-8 and TNF-α and ultimately contributing to gingival health via immunosurveillance [26]. Additionally, this commensal improved paracellular barrier function by increasing the expression of genes encoding the tight junction components ZO-1, ZO-2, JAM-A, and occludin [39].

To date, it remains unclear if commensal bacteria can interfere with the ability of *P. gingivalis* to invade host cells. If so, this means that the commensal bacteria promote a protective effect in the context of pathogenic invasion. The aim of this study is to determine whether commensal bacteria can slow *P. gingivalis* invasion into host cells in sequential co-cultures. We are investigating the effect of a pre-exposure to commensal bacteria on oral epithelial cells to determine if that interaction protects epithelial cells from a subsequent *P. gingivalis* invasion. We hypothesize that the interaction between commensal bacteria and oral epithelial cells will directly hinder *P. gingivalis* invasion.

## 2. Materials and Methods 

### 2.1. Cell Line and Bacterial Cultures

The immortal human oral keratinocyte OKF6/Tert cell line was kindly provided by Dr. Gill Diamond from the University of Florida, College of Dentistry (Gainesville, FL, USA) [40]. Cells were cultured in tissue culture plates at 37 °C in 5% CO_2_ (standard conditions) with complete Keratinocyte Serum-Free Growth Medium (KSFM) prepared following the methods described by Brice et al. [40]. Briefly, KSFM base medium plus 30 μg/mL of bovine pituitary extract, 0.3 mM calcium chloride, 1 mM glutamine, and 100 U/mL of penicillin and streptomycin was used. Cells were sub-cultured every 4 to 6 days. All experiments were conducted between passages 21 and 37.

*Streptococcus gordonii* DL1, *Streptococcus intermedius* 0809, *Streptococcus mitis* UF2, and *Streptococcus oralis* SK139 were grown in Brain Heart Infusion Broth (BHI) with 5 µg/mL hemin or in BHI agar at 37 °C in 5% CO_2_ (standard conditions). All four commensal species were kindly provided by Dr. Robert Burne from the University of Florida, College of Dentistry (Gainesville, FL, USA). *Porphyromonas gingivalis* 33277 was kindly provided by Dr. Richard Lamont at the University of Louisville, School of Dentistry (Louisville, KY, USA). *P. gingivalis* 33277 was grown in Trypticase soy broth with yeast extract (1 mg/mL), hemin (5 μg/mL), and menadione (1 μg/mL) (TSBY) or in blood agar with menadione (1 μg/mL) anaerobically at 37 °C in a Bactron 300 anaerobic incubator from Sheldon Manufacturing, Inc. (Cornelius, OR, USA).

### 2.2. Bacterial Growth Curves

Starter cultures of *S. gordonii* DL1, *S. mitis* UF2, *S. intermedius* 0809, and *S. oralis* SK139 were grown in BHI media overnight at 37 °C in 5% CO_2_. The following day, starter cultures were adjusted to OD_595nm_ = 1.0, and 1% inoculum was used to grow new cultures in BHI and KSFM without antibiotics. The cultures were incubated at 37 °C in 5% CO_2_, and absorbance was read every 2 h for 12 h and at 24 h post-inoculation. 

### 2.3. Streptococcal Cytotoxicity Assay

OKF6/Tert cells were grown in 12-well plates with 1 mL of complete KSFM/well to at least 90% confluency. Streptococci were grown to late exponential phase, and cultures of each species were adjusted to OD_595nm_ = 1.0. Bacteria were diluted separately in antibiotic-free KSFM. OKF6/Tert cells were washed three times with 1 mL of sterile PBS per well to remove excess antibiotics. Bacteria in their spent media were diluted in antibiotic-free KSFM to ensure that the microbes and their products were involved in all assays, mimicking in vivo interactions as close as possible. After diluting, all streptococcal strains and their extracellular products were added to OKF6/Tert cells at a multiplicity of infection (MOI) of 100 in a final volume of 1 mL in separate wells. In addition, matching volumes of filtered-sterile spent BHI media were diluted in KSFM, or KSFM alone was added to OKF6/Tert cells as controls for cytotoxicity. All co-cultures and corresponding controls were then incubated for 1 or 2 h post-infection under standard conditions. Then, OKF6/Tert cells were washed three times with 1 mL of sterile PBS per well to remove unbound streptococci, and an antibiotic protection assay was performed. Briefly, OKF6/Tert cells were incubated for an hour with 1 mL of KSFM containing 300 µg/mL of gentamicin and 200 µg/mL of metronidazole to kill extracellular bacteria [27,28,29,41,42]. Then, cultures were incubated for 48 h post-infection in complete KSFM (containing penicillin and streptomycin) under standard conditions. To assess cellular morphology during and after host–bacteria interactions, images of the keratinocyte monolayers were acquired at 200X magnification using a Nikon Eclipse TE2000-U inverted microscope equipped with a Nikon Digital Sight DS-Fi1 camera and NIS Elements Imagine Software (Nikon Instruments Inc, Melvin, NY, USA) at indicated time-points. After 48 h post-infection, cells were trypsinized and mixed with an equal volume of trypan blue, which diffuses inside dead cells, thus staining them blue under the light microscope. Live (unstained) cells were counted using the hemocytometer and light microscopy. 

### 2.4. Streptococcal Adherence and Invasion Assays

Co-cultures of OKF6/Tert cells with either *S. gordonii* DL1 or *S. intermedius* 0809 at MOI = 100 were incubated for 1 h as above. Excess bacteria were then washed three times with 1 mL of PBS per well, and OKF6/Tert cells were either lysed with 1 mL of sterile diH_2_O per well or subjected to an antibiotic protection assay as described above. After the hour of killing, OKF6/Tert cells were washed and lysed as above. Lysates before and after the antibiotic protection assay were plated on BHI agar for CFU counts to quantify commensal bacteria adherence and invasion, respectively.

### 2.5. P. gingivalis Invasion Assays

OKF6/Tert cells were incubated for 1 h with either *S. gordonii* DL1 or *S. intermedius* 0809 as above. Then, cells were washed three times with 1 mL of PBS per well to remove excess commensal bacteria. To ensure the streptococci that adhered to the OKF6/Tert cells remained alive, no antibiotic protection assay was performed at this point. The *P. gingivalis* 33277 overnight culture was adjusted to OD = 1.0, diluted in antibiotic-free KSFM, and added to cells pre-exposed to streptococci at MOI = 100. Equal quantities of *P. gingivalis* 33277 were also added to OKF6/Tert cells that were not exposed to streptococci (control). Co-cultures were incubated for 30 min under standard conditions [28,43]. Then, cells were washed three times with 1 mL of PBS per well, and an antibiotic protection assay was performed to kill all extracellular bacteria, as described above. Cells were washed and lysed as indicated above. Lysates were serially diluted in sterile PBS, plated on blood agar plates with menadione, and incubated for about 5 days anaerobically before counting *P. gingivalis* colonies.

### 2.6. Streptococcal Inhibition of P. gingivalis Growth

Growth inhibition experiments were conducted following the methodology of Herrero et al. [20]. *Escherichia coli* does not have any effect on *P. gingivalis* growth [20] and therefore was used as a negative control for inhibition experiments. Late exponential phase cultures of *S. gordonii* DL1, *S. intermedius* 0809, and *E. coli* OP50 grown in BHI (OD = 1.0) were diluted to 1:100 in TSBY. Ten microliters of each strain were plated on separate blood agar plates with 1 μg/mL of menadione and allowed to dry. Bacteria were cultured for 24 h. *P. gingivalis* was grown overnight in TSBY anaerobically, the OD was adjusted to 1.0, the cultures were diluted 1:10 in TSBY, and 10 µL were plated on the same blood agar approximately 1 mm away from the spots of the already existing strains. Bacteria were cultured for about three days anaerobically, and the plates were photographed to observe *P. gingivalis* growth patterns. 

### 2.7. Statistical Analyses

Every experiment was repeated at least three times. Figures represent the mean and standard deviation of the data. T-tests and ANOVAs were performed, and *p* values < 0.05 were noted as significant. 

## 3. Results

### 3.1. KSFM Supports Growth of Commensal Bacteria

**Figure 1:**
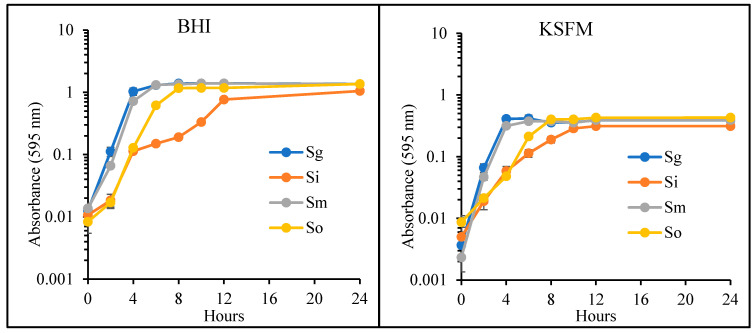
Growth of commensal bacteria in Brain Heart Infusion Broth (BHI) and Keratinocyte Serum-Free Growth Medium (KSFM). *S. gordonii* (Sg), *S. intermedius* (Si), *S. mitis* (Sm) and *S. oralis* (So) were grown in KSFM and BHI. Absorbance was measured at indicated time-points (*n* = 3).

In order to compare growth patterns, all commensals were grown in BHI and KSFM without antibiotics. As shown in Figure 1, *S. gordonii* had the most rapid growth in BHI, followed by *S. mitis*, *S. oralis* and *S. intermedius*. *S. gordonii* and *S. mitis* entered the exponential phase of growth by 2 h and continued growing, reaching the late-exponential phase at 4 h. For these species, the stationary phase lasted from Hour 4 to the end of the experiment (Figure 1). *S. oralis* grew exponentially from Hours 2 to 6 and then continued into the stationary phase for the remainder of the experiment (Figure 1). *S. intermedius* reached the end of exponential phase at 12 h. Similar growth patterns were observed in KSFM. However, for all species, absorbance values throughout the experiment remained below 0.5 in this medium. Once again, *S. gordonii* and *S. mitis* grew the fastest in KSFM compared to the other two species (Figure 1). *S. intermedius* also experienced the slowest growth in KSFM. Our data indicate that these four streptococci are not harmed in KSFM. This cell culture medium supports their growth but to a limited absorbance value when compared to growth in BHI broth (Figure 1).

### 3.2. OKF6 Cell Viability after Commensal Challenge

**Figure 2:**
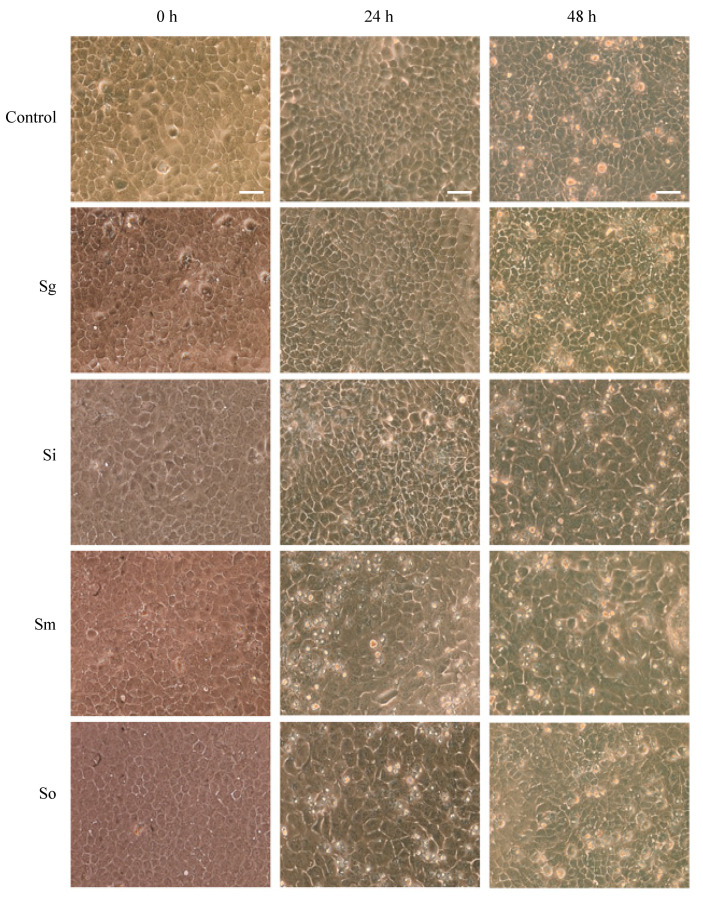
OKF6/Tert cellular morphology after exposure to *S. gordonii* (Sg), *S. intermedius* (Si), *S. mitis* (Sm), and *S. oralis* (So) at MOI = 100 for 1 h of interaction. Light microscopy images were obtained at a magnification of 200×. Bars = 100 μm. Representative images from three independent experiments.

**Figure 3:**
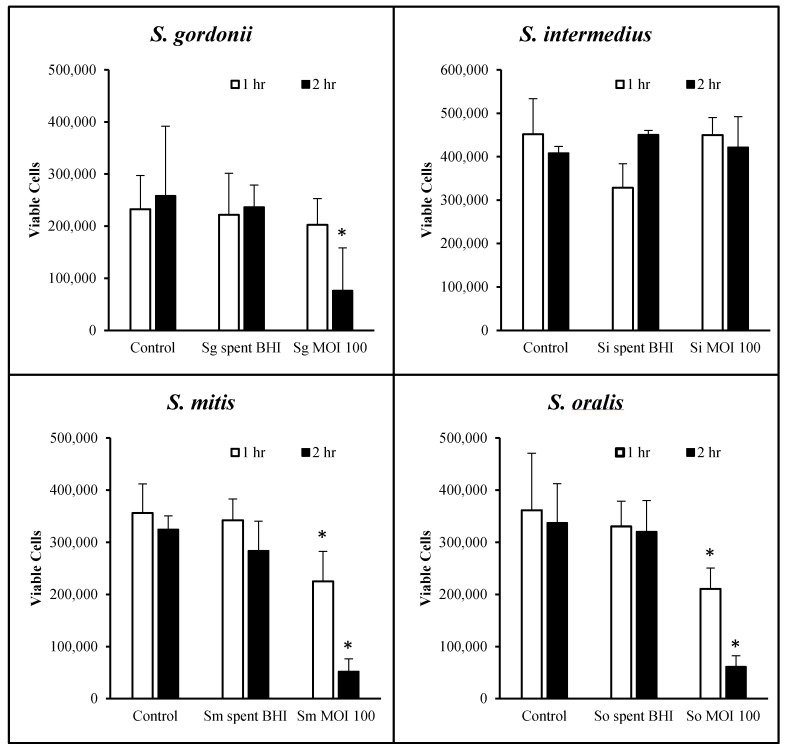
Viability of OKF6/Tert cells following exposure to commensal streptococci. Cell viability reflects the number of live OKF6 cells two days after exposure to *S. gordonii* (Sg), *S. intermedius* (Si), *S. mitis* (Sm), or *S. oralis* (So) or their spent media. Live cells were quantified using the trypan blue exclusion assay. Graphs represent the average and SD of four independent experiments. * = *p* < 0.05 compared to control.

Prior to any further studies, we determined the periods of time for which OKF6/Tert cells can withstand each of the oral streptococci at MOI = 100. Figure 2 shows the cellular morphology at 0, 24 and 48 h after exposure with all four commensals for 1 h of host–bacteria interactions in 24-well plates. The results show that the cells were nearly 100% confluent at the time of exposure to bacteria and remained at high confluency until 48 h post-exposure. The cellular morphology seems to be intact, but some cellular changes begin to appear after exposure to *S. mitis*, *S. intermedius* and *S. oralis* at 24 h of incubation (Figure 2). However, by 48 h of incubation, cellular morphologies in all conditions seem comparable to each other and to control. To assess the viability of cells, trypan blue exclusion assays were performed. *S. gordonii* did not cause significant OKF6/Tert cell death after 1 h of bacterial exposure. However, this commensal did cause significant cell death after 2 h of exposure (Figure 3). Cells remained viable following challenge by *S. intermedius* at a MOI of 100 for up to 2 h (Figure 3). Exposure to *S. oralis* and *S. mitis* resulted in significant cell death after 1 and 2 h compared to untreated controls (*p* < 0.05). The effect of these two commensals is time-dependent as more cell death is observed over prolonged periods of challenge. In every case, OKF6 cells remained viable when exposed, for up to 2 h, to the spent BHI media of any of the four strains. These results indicate that, under the conditions tested, cytotoxicity only occurs when three of the four streptococci are present and physically interacting for at least 2 h with the OKF6/Tert cells. Based on these results, the remainder of the study was performed using only *S. gordonii* and *S. intermedius* for up to 1 h of interaction.

### 3.3. Commensal Bacteria Adherence Assay

**Figure 4:**
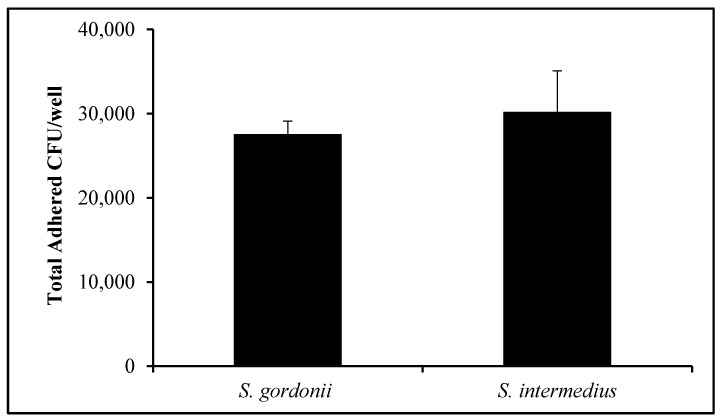
Adherence of commensal bacteria. Quantification of oral commensal streptococci CFUs after adherence to OKF6/Tert cells (MOI = 100) in 24-well plates. Graph represents the average and SD of six independent experiments.

OKF6/Tert cells were exposed to *S. gordonii* and *S. intermedius*. Figure 4 displays the total number of CFUs per well for both streptococci after incubation for 1 h. Following thorough washing, 27,574 +/− 1539 CFUs of *S. gordonii* per well were recovered. Similarly, 30,203 +/− 4883 CFUs of *S. intermedius* were quantified after adherence to OKF6/Tert cells (Figure 4). This suggests that, when contrasting the number of OKF6/Tert cells per well in Figure 3 to the number of adhered CFUs per well, roughly one streptococcal CFU of either of these species adheres to every 10 to 15 cells on average (Figure 4). In addition, following antibiotic protection assays, an average of 96 or 99 CFUs of *S. gordonii* and *S. intermedius,* respectively, were found for every 100,000 host cells (data not shown). These results suggest that these two oral commensal streptococci invade at extremely low rates or not at all.

### 3.4. P. gingivalis Invasion Post-Commensal Co-Cultures

**Figure 5:**
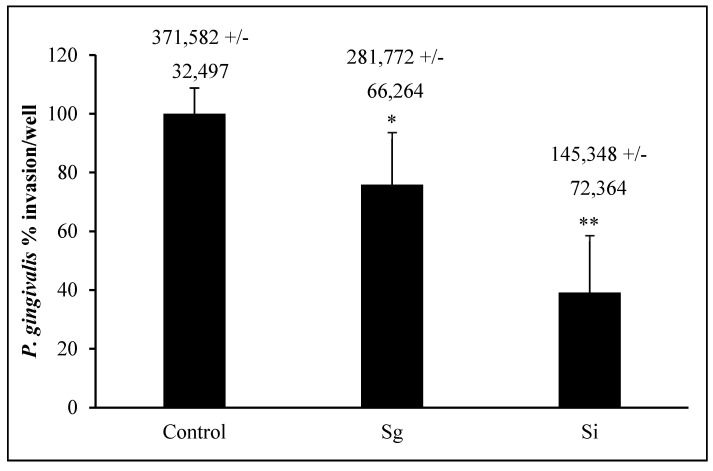
Invasion of *P. gingivalis* following exposure to commensal bacteria. Percentage of *P. gingivalis* CFUs (absolute values +/− SD above each bar) that invaded OKF6/Tert cells after exposure to *S. gordonii* (Sg) or *S. intermedius* (Si). All bacteria were added to OKF6/Tert cells at MOI = 100. Graph represents the average and SD of four independent experiments. * = *p* < 0.05 compared to control. ** = *p* < 0.05 compared to control and Sg.

*P. gingivalis* 33277 invasion into OKF6/Tert cells is around 370,000 bacteria into about 300,000 to 400,000 cells/well, which makes the bacterium/cell ratio around 1:1 (Figure 5). This result agrees with the results obtained by others [28,41,42,43]. *S. gordonii* and *S. intermedius* pre-treatments of OKF6/Tert cells reduced *P. gingivalis* invasion to about 75% and 40%, respectively (Figure 5). In addition, *S. intermedius* treatment reduced the amount of *P. gingivalis* invasion into OKF6/Tert cells significantly less than *S. gordonii* treatment (*p* value < 0.05). Our results indicate that during the host–bacteria interactions of OKF6/Tert cells with either commensal, *P. gingivalis* invasion is significantly reduced.

### 3.5. Commensal Antagonistic Properties against P. gingivalis

**Figure 6:**
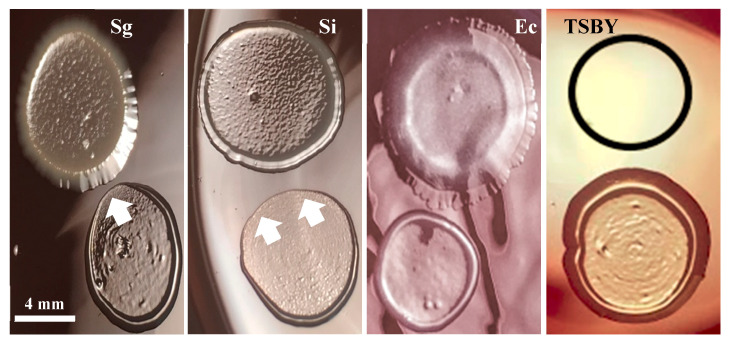
*S. gordonii* and *S. intermedius* antagonism against *P. gingvalis.* All bacteria were diluted in TSBY before plating. Spots for *S. gordonii* (Sg), *S. intermedius* (Si), *E. coli* (Ec), diluted 1:100, or TSBY are shown on the top of each image. Spots for *P. gingivalis*, diluted 1:10, are shown at the bottom of every image. White arrows point at areas of *P. gingivalis* spots with growth inhibition. Representative images from three independent experiments.

Figure 6 shows *S. gordonii* and *S. intermedius*’s inhibiting effects on *P. gingivalis*. *E. coli*, which has already been shown to have no effect on the growth of *P. gingivalis* [20], and TSBY (outlined in the black circle) were used as negative controls for inhibitory effects. Both oral commensal streptococci show antagonistic relationships with *P. gingivalis*, as indicated by the white arrows pointing at the areas of growth inhibition. Note that these areas are directly in front of the oral commensal streptococci spots. These results suggest that *S. gordonii* and *S. intermedius* produce inhibitory substances that diffuse through the medium and inhibit *P. gingivalis* growth. This antagonism may be related to the commensal streptococci’s ability to decrease *P. gingivalis* invasion into the OKF6/Tert cells (Figure 5).

## 4. Discussion

Overall, our study demonstrates that commensal bacteria can provide a protective effect for the host in the context of pathogenic invasion. The specific mechanisms of commensal bacterial protection remain unknown. However, these mechanisms may serve as additional therapeutic methods to treat PD.

KSFM was used as the vehicle for bacteria to establish the MOI throughout the study. KSFM does support the growth of all commensals tested, albeit at a much slower pace than BHI. Therefore, some bacterial growth is expected to occur during the interaction with the host, and this may cause the MOI values to deviate slightly throughout the experiments. Interestingly, *S. gordonii* and *S. intermedius* are the commensal strains that grow the fastest and slowest, respectively (Figure 1). However, neither of these species appears to be harmful to the host within the first hour of host–bacteria interactions (Figure 3). Furthermore, even though *S. intermedius* can be expected to exist in lower quantities than *S. gordonii*, by the end of the hour of host–bacteria interactions, both species were bound to the host at similar levels (Figure 4). Nonetheless, *S. intermedius* is the strain that confers the best protection to the host cells (Figure 5).

Figure 2 shows that the cellular morphology of OKF6/Tert cells does not seem to change significantly after one hour of interactions. However, *S. oralis* and *S. mitis* lead to the substantial death of OKF6/Tert cells after the same treatment (Figure 3). The spent media of all commensals tested, on the other hand, did not harm the host cells, which may suggest that the death of OKF6/Tert cells requires direct contact or the activity of the species near the host. We tested the possibility of streptococcal invasion into OKF6/Tert cells (data not shown) and found extremely low levels to no invasion at all. We propose that the commensal strains *S. gordonii* and *S. intermedius* do not invade OKF6/Tert cells, which is in agreement with the little to no invasion observed with *S. gordonii* by others [26]. Interestingly, since *S. mitis* and *S. oralis* are cytotoxic, it is unclear if cells exposed to these bacteria may be more susceptible to *P. gingivalis* invasion or how this dynamic may affect the development of PD.

The methodology of CFU counts was used primarily because of its reliability in counting the live bacteria harvested at the end of every experiment. Quantitative PCR can also take account of the total bacteria per well but with the limitation of including live and dead bacteria. For example, commensal bacteria that are bound to OKF6/Tert cells and undergo antibiotic protection assays will still contain DNA, which will still be amplified during the qPCR assay. Similarly, in fluorescence-based microscopy techniques, fluorescent labels would not distinguish between live and dead bacteria, and therefore, the microscopic images would present all bacteria associated with OKF6/Tert cells. Because of the limitations of all those other techniques, we chose CFU counts as a dependable method for measuring live bacteria only.

Streptococci CFUs do not necessarily reflect one bacterium, but often a chain of bacteria that may include 10 to 20 streptococci. This explains the CFU/cell ratio of roughly 1 CFU corresponding to roughly 10 to 15 host cells, which means that a given chain of commensal bacteria could have been bound to one of every twelve cells on average. It becomes plausible that several commensal bacteria in a chain are blocking potential *P. gingivalis* receptors, decreasing pathogenic invasion into the host cells. However, this does not explain the decrease in invasion in the neighboring host cells. In order to decrease invasion, other mechanisms must be in place to prevent *P. gingivalis* from entering these host cells. Such mechanisms of streptococcal host protection from *P. gingivalis* invasion remain unknown. 

Figure 6 demonstrates that both *S. gordonii* and *S. intermedius* have antagonistic relationships with *P. gingivalis*. These results are in concordance with the results of Herrero et al., where *S. gordonii* inhibited *P. gingivalis* growth via the production of hydrogen peroxide. Herrero et al. report the production of peroxide by several species of oral commensal bacteria, inhibiting the growth of pathogens including *P. gingivalis*, *Aggregatibacter actinomycetemcomitans*, and *Prevotella intermedia* [20]. In accordance, we also report *S. gordonii* and *S. intermedius*’s inhibition of *P. gingivalis* growth (Figure 6), which could suggest a putative mechanism by which peroxide production may deter *P. gingivalis* from approaching the host. An additional mechanism could be the streptococcal binding to and concealing of common host receptors. In order to address this point, studies aiming to (i) identify the surface molecules on *S. intermedius* that serve as anchoring agents for host epithelial tissues and (ii) add these surface molecules to the host in competition assays with *P. gingivalis* may be considered. Molecules from a commensal species that prevent the colonization and invasion of *P. gingivalis* may be used to enhance therapies for PD. Lastly, in healthy individuals who maintain good oral health, commensal streptococci create a beneficial oral biofilm that contributes to the host immune response [19,44]. Therefore, it is possible that these commensal species are protective against the invasion of pathogens in vivo.

Our study agrees with other studies where commensal bacteria work in cooperation with the host to inhibit the pathogenesis of disease-causing bacteria [45,46]. For example, several species of commensal gut bacteria show protective effects against airway infections by *Klebsiella pneumoniae* and *Streptococcus pneumoniae* [47]. An interesting study demonstrates that *Akkermansia muciniphila* from the gut decreases *P. gingivalis*-based periodontitis in vivo [48]. Furthermore, *S. gordonii* itself promotes the improvement of the epithelial barrier (tight junctions) [39], as well as the expression of cytokines, human defensins, and other immunological mediators [26,44,49,50]. Our results align well with other studies demonstrating that commensal bacteria can slow pathogenic activity.

Cytokine production could be an important parameter to measure. However, we feel that it falls outside of the aim of this study because cytokines are relevant to the cellular immune response. Our study is most concerned with the interactions between commensal bacteria and the host leading to protection from invasive *P. gingivalis*. Several other studies address this inquiry [26,38,44,51,52,53,54]

In conclusion, we have observed that *S. gordonii* and *S. intermedius* do not cause cell death when compared to the other two commensal species tested. Both species adhere to our oral epithelial cell model and significantly decrease the invasion of periodontopathogen *P. gingivalis,* offering protection to the host. *S. intermedius* reduced *P. gingivalis* invasion by more than 60%. The mechanisms of this protective effect are still unknown.

The invasion of *P. gingivalis* plays a large role in the initiation and progression of PD and therefore the oral health of individuals [55]. Oral health is important in maintaining overall health [15,56,57,58]. At a higher risk for PD, Americans 65 years of age and older may experience tooth loss, pain, discomfort, the inability to properly chew and swallow food, and ultimately poor nutrition [2]. The role of *P. gingivalis* in systemic diseases further heightens the gravity of this pathogen. Our work is innovative in the context of using the oral commensal microbiota against pathogens such as *P. gingivalis* in an effort to decrease the chances of periodontal disease.
